# A rare case of encephalomyelitis caused by *Candida parapsilosis*

**DOI:** 10.1111/cns.14062

**Published:** 2022-12-19

**Authors:** Jing Li, Jialing Liu, Shu Jia, Ruihua Li, Xiaoting Chang, Yubing Sun, Yongzhong Lin

**Affiliations:** ^1^ Department of Neurology The Second Affiliated Hospital of Dalian Medical University Dalian China; ^2^ Department of Clinical Laboratory The Second Affiliated Hospital of Dalian Medical University Dalian China

## 1 INTRODUCTION

Pathogens that cause fungal infections in the central nervous system (CNS) include the *Cryptococcus*, *Aspergillus*, *Histoplasma capsulatum*, and *Candida* species, among which the *Candida* species are uncommon. It is even rarer for the *Candida* species to invade the spinal cord. Here, we present the first case of encephalomyelitis caused by *Candida parapsilosis*. Cerebrospinal fluid (CSF) metagenomic next‐generation sequencing (mNGS) and CSF serum (1,3)‐β‐D‐glucan (BDG) were used to aid in the diagnosis, and serum BDG was used to track the efficacy of antifungal therapy.

## 2 CASE PRESENTATION

A 57‐year‐old male was admitted to the hospital because of dizziness, nausea, and weakness of the left limbs for 11 h. He had a history of syphilis but no history of hypertension, diabetes, or atrial fibrillation. On admission, neurological examination revealed a muscle power grade of 2/5 in the left upper and lower limbs, positive Babinski sign on the left side, urine retention, and constipation. Brain computed tomography revealed no abnormalities. The patient developed chills, shivering, and fever (38.1°C) on the first night. Laboratory findings revealed a white blood cell (WBC) count of 8.62 × 109/L, with 92% neutrophil percentage. He was then started on empirical broad‐spectrum antibacterial therapy with piperacillin‐tazobactam (4.5 g Q8h) (Detailed diagnosis and treatment flow chart was shown in Appendix S1).

**FIGURE 1:**
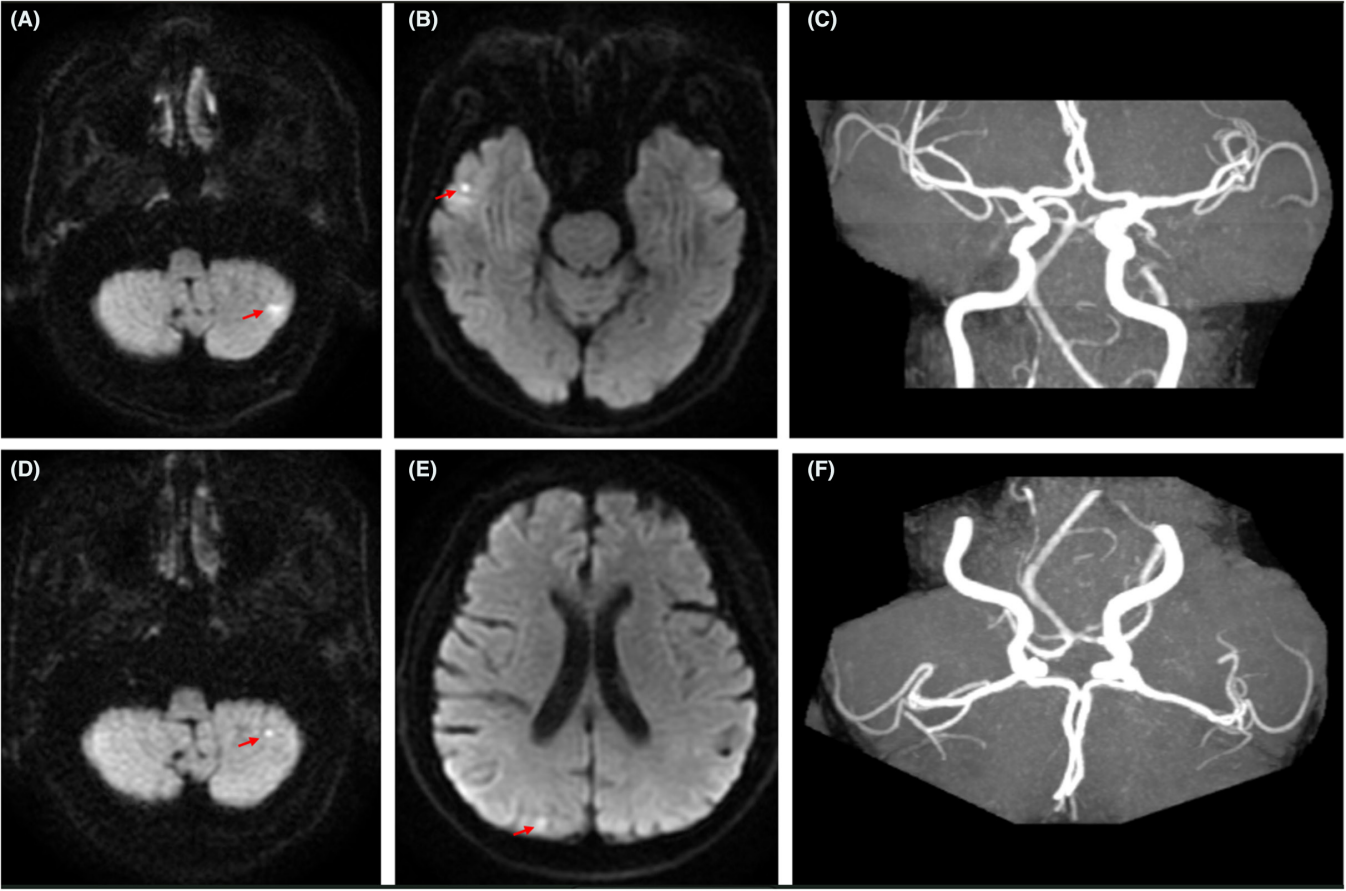
Brain MRI. (A, B) DWI of the brain on day 3 showed patchy high signal intensities in the right temporal lobe and left cerebellum. (C, F) No obvious blood vessel obstruction or stenosis was observed by magnetic resonance angiography. (D, E) DWI of the brain on day 34 revealed slightly hyperintense signals in the left cerebellum and right occipital lobe. (magnetic resonance imaging, MRI; Diffusion‐weighted imaging, DWI)

**FIGURE 2:**
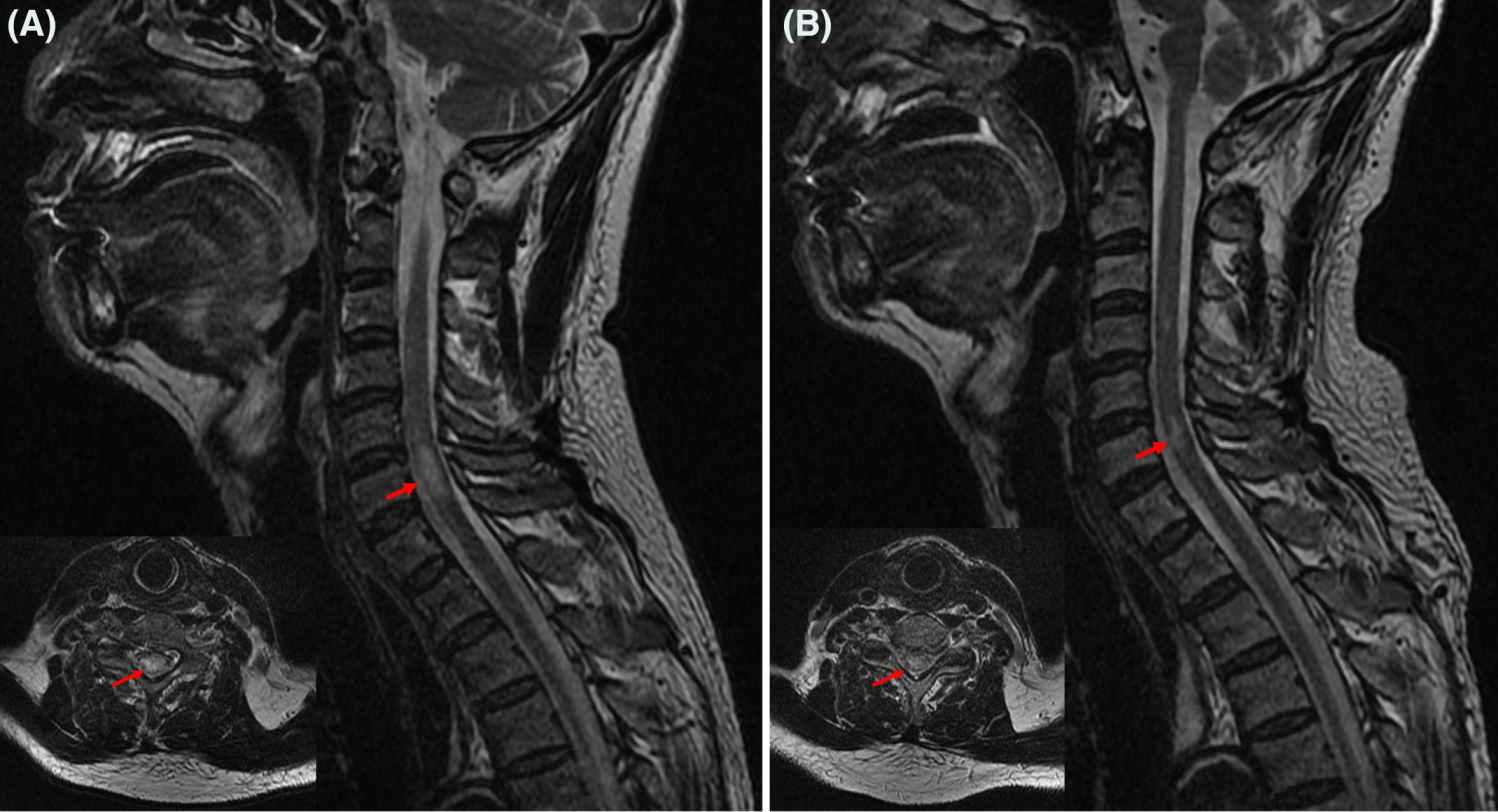
Cervical spine MRI. (A) T2‐weighted MRI of the cervical spine on day 9 presented an intramedullary high signal. (B) T2‐weighted MRI of the cervical spine on day 34 showed a significantly absorbed abnormal signal. (magnetic resonance imaging, MRI)

Diffusion‐weighted imaging (DWI) of the brain showed patchy high signal intensities in the right temporal lobe and left cerebellum. No obvious blood vessel obstruction or stenosis was observed on magnetic resonance angiography (Figure 1A–C,F). Rapid serum plasma reagin was positive for syphilis. CSF analysis indicated a light‐yellow fluid with a pressure of 110 mm H_2_O, WBC count of 77 cells/μl (78% lymphocytes), glucose of 1.3 mmol/L, protein of 3.34 g/L (Table 1), and negative rapid plasma reagin test results. On day 6, the peripheral blood culture was positive for *Candida parapsilosis*, with a BDG serum concentration of 338.34 pg/ml (Table 2). Subsequently, fluconazole (400 mg/day; 800 mg on the first day) antifungal therapy was initiated, and piperacillin‐tazobactam was discontinued. However, he developed right upper‐ and lower‐limb weakness and general numbness on day 9. Through neurological assessment, we found left upper and lower limb muscle strength of grade 2, right upper and lower limb distal muscle strength of grade 4, bilateral Babinski sign (+), Hoffman's sign on the left side (+), and hypesthesia below the clavicle. Reexamination of the head DWI revealed a dotted, slightly high‐signal shadow in the right occipital lobe. Magnetic resonance imaging (MRI) of the cervical spine demonstrated an intramedullary high‐signal shadow at the level of the cervical vertebrae 3, 4, and 6 on T2 weighted images (Figure 2A). The lumbar puncture was repeated, and the CSF profile revealed light‐yellow fluid with a pressure of 100 mm H_2_O, 120 white blood cells/μl (75% lymphocytes), 1.3 mmol/L glucose, 2.83 g/L protein, and >600 pg/ml BDG (Table 1). We assumed that the fungus had invaded the CNS and treated the patient with amphotericin B (5 mg/kg/day).

**TABLE 1:** The results of CSF samples obtained by lumbar puncture at different time points after admission

Time (Day)	WBC (μl)	L percentage (%)	Glucose (mmol/L)	Protein (g/L)	Bacterial culture	Fungal culture	BDG (pg/ml)
5	77	78	1.30	3.34	Negative	‐	‐
9	120	75	1.30	2.83	Negative	*C. parapsilosis*	>600
16	110	89	1.40	2.65	Negative	*C. parapsilosis*	>600
23	158	95	1.80	3.64	Negative	Negative	>600
27	90	90	1.70	2.87	Negative	Negative	>600
30	87	98	1.90	2.80	Negative	Negative	>600
33	110	96	1.90	2.25	‐	‐	‐
35	54	96	2.10	1.75	‐	‐	‐
38	90	92	2.30	1.23	‐	‐	>600
41	115	97	2.20	1.14	Negative	Negative	>600
43	35	97	2.20	1.04	‐	‐	‐
47	30	85	2.40	1.00	‐	‐	‐
50	22	99	2.30	0.98	Negative	Negative	>600
52	20	87	2.50	0.90	Negative	Negative	‐
54	17	70	2.60	0.84	‐	‐	>600

**TABLE 2:** The results of BDG in serum samples at different time points after admission

Time (Day)	3	6	16	20	26	28	35	43	50	Day 54
BDG (pg/ml)	338.34	230.93	212.22	336.32	374.21	280.25	272.52	223.34	150.91	144.20

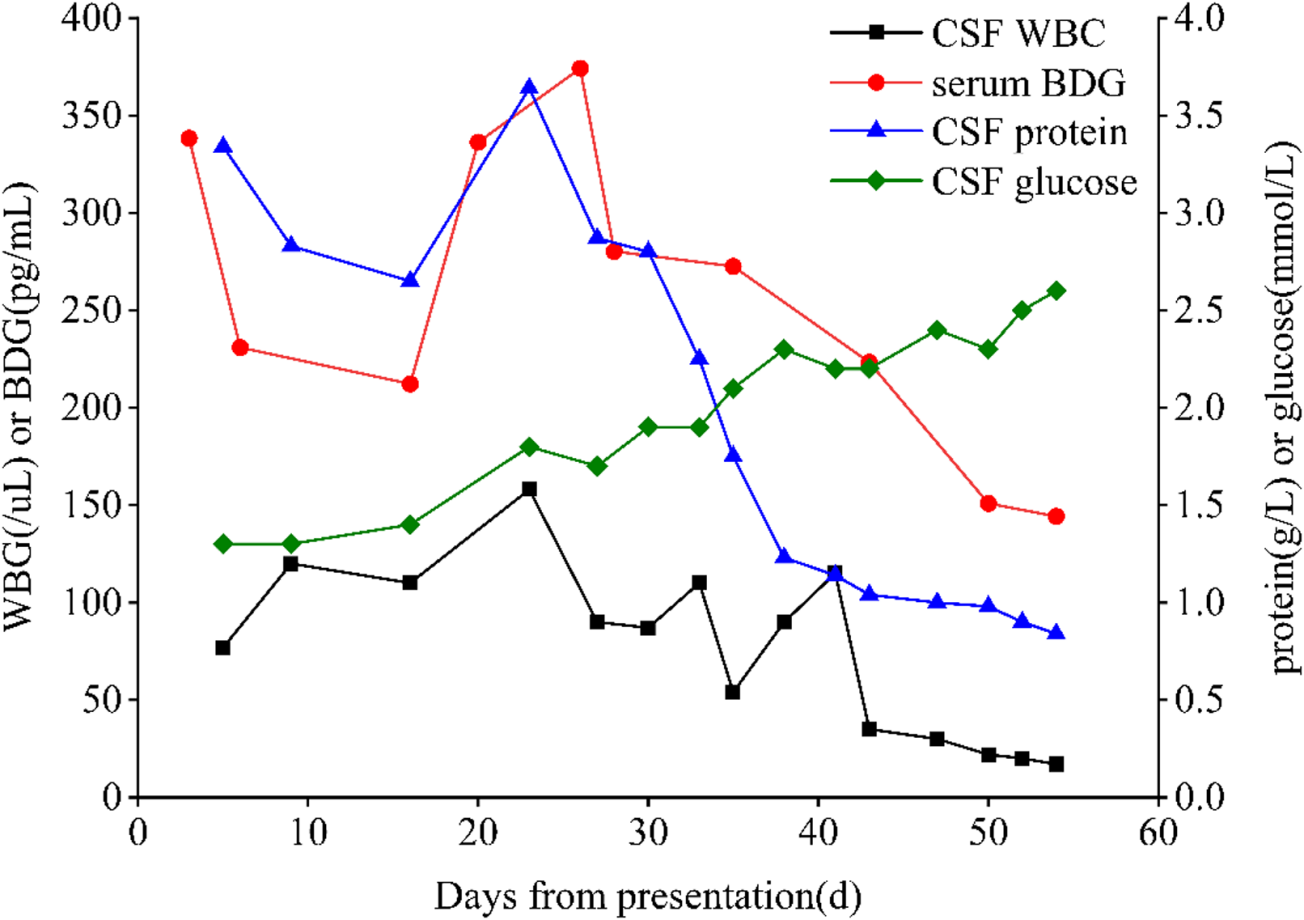

After five days, no significant decrease in body temperature or improvement in symptoms or signs was observed. *Candida parapsilosis* was detected in the CSF culture and was sensitive to common antifungal drugs, such as amphotericin B and fluconazole. CSF mNGS showed the presence of *Candida parapsilosis* with 307 reads and *Pseudomonas aeruginosa* with 50 reads. The fluconazole dose was adjusted to 800 mg daily, and antibacterial therapy with meropenem (2 g Q8h) was initiated. Nevertheless, on day 23, the patient's condition deteriorated, with exacerbation of CSF protein and WBC and elevation of the serum BDG concentration. Therefore, he was treated with intrathecal injections of amphotericin B (0.1 mg initially, gradually increased to 0.5 mg; 2–3 times per week) and dexamethasone (2 mg). His body temperature returned to normal and remained normal for more than three days on day 34. Moreover, the results of serum BDG and the CSF routine test results gradually improved. Repeated DWI of the brain showed slightly hyperintense signals in the left cerebellum and right occipital lobe (Figure 1D,E). T2‐weighted MRI of the cervical spine revealed that the abnormal signal was significantly absorbed (Figure 2B). Therefore, the meropenem treatment was discontinued. By the 54th day, the motor weakness had improved. Physical examination indicated that the muscle strength of the left upper and lower limbs was grade 3, while that of the right limbs was the same as before. The serum BDG level declined to 144.20 pg/ml. The CSF was colorless and transparent, with a pressure of 102 mm H_2_O, WBC count of 17 cells/μl (97% lymphocytes), glucose 2.6 mmol/L, protein 0.84 g/L, and BDG >600 pg/ml. The intrathecal injections of amphotericin B and dexamethasone were discontinued. The patient was discharged from the hospital on day 60 and administered oral fluconazole (800 mg/day).

## 3 DISCUSSION

There are only a few reports of *Candida* infections in the spinal cord.^1^ To the best of our knowledge, encephalomyelitis caused by *Candida parapsilosis* has never been reported. In our case, there were also pathological changes in the brain, in addition to the lesion located in the spinal cord.

Fungal infections usually occur in immunocompromised individuals. The numbers of CD4+ and CD8+ T lymphocytes, CD4+/CD8+ ratio, and other immune indicators were measured to evaluate the patients' immune responses. However, these results were normal. In addition to immunodeficiency, CNS fungal infections have also been observed in patients with fungal infections of tissues adjacent to the skull base, a history of intracranial surgery, and antibiotic abuse.^2^ The above risk factors were ruled out through relevant auxiliary examinations and consultations with the relevant departments. Considering that the pathogens in the patient's blood and CSF were both *Candida*, we considered that the CNS fungus was derived from the blood. The source of candidemia was unknown in 52.5% of the episodes.^3^ Unfortunately, this was only one of them.

CSF mNGS has numerous advantages, including high sensitivity, short processing time, and broad‐spectrum detection. It has significantly improved the diagnostic rate of CNS infections and could aid in the diagnosis of cryptic CNS infections.^4^, ^5^ In this case, *Candida parapsilosis* and *Pseudomonas aeruginosa* were detected in the initial CSF mNGS. Only *Candida parapsilosis* was detected in multiple blood and CSF cultures. We considered that *Pseudomonas aeruginosa* co‐infection was not excluded when treating *Candida*; therefore, meropenem was added for antibacterial treatment. However, its therapeutic effect was unsatisfactory. Upon treatment with antifungal drugs and dexamethasone, the patient's clinical symptoms improved, the lesions appeared to recede on MRI, and CSF leukocyte and protein levels decreased significantly. After discussion with many experts, it was believed that *Pseudomonas aeruginosa* might be a false positive. The reasons for this are as follows: (1) Additional anti‐*Pseudomonas aeruginosa* therapy was administered; however, no significant improvement in the state of illness was observed. (2) Repeated blood and CSF cultures for *Pseudomonas aeruginosa* were negative. (3) *Pseudomonas aeruginosa* is a common contaminant that may come from hospitals or laboratories. Our case suggests that caution should be exercised when interpreting mNGS results. To minimize false positives, we should perform a comprehensive evaluation and judgment in combination with the patient's condition and other laboratory test results.

Previous studies have demonstrated that CSF BDG can assist in the diagnosis of CNS fungal infections.^6^ However, only a few reports of CNS *Candida* infections are available, in which CSF BDG levels were greater than or equal to 500 pg/ml.^7^, ^8^, ^9^ In this report, the CSF BDG value was consistently higher than 600 pg/ml, which could help diagnose CNS *Candida* infection. CSF cultures cannot be quantified and require a long turnaround time. In addition, molecular methods such as mNGS are complicated and costly. Therefore, a valid indicator for monitoring the effectiveness of antifungal therapy remains unclear. In our case, the serum BDG level gradually decreased as the patient's condition improved, correlating with CSF protein, glucose, and WBC. This implied that serum BDG levels were useful in therapeutically monitoring CNS *Candida* infections, which is consistent with the results of Salvatore et al.^10^

In conclusion, we report the first case of encephalomyelitis caused by *Candida parapsilosis*. CSF culture can provide drug sensitivity results, mNGS can efficiently and broadly screen pathogens, CSF BDG can play an auxiliary role in diagnosis, and serum BDG can be used to track the efficacy of antifungal therapy. We recommend that the above diagnostic methods should be individually selected according to the situation of patients suspected of having CNS *Candida* infections.

## 4 AUTHOR CONTRIBUTIONS

JL performed the data acquisition and wrote the manuscript. JLL, SJ, RHL, XTC and YBS collected radiological images. YZL reviewed the manuscript and provided critical revision. All authors contributed to the study and approved the submitted version.

## 5 FUNDING INFORMATION

This study was supported by Basic Research on the Application of Dalian Innovation Fund in Dalian(2020JJ26SN054).

## 6 CONFLICT OF INTEREST

The authors declare no conflict of interest.

7

## Supporting information


Appendix S1

## Data Availability

The original contributions presented in the study are included in the article. Further inquiries can be directed to the corresponding author.
