# Supplementary figures and images for: A rare case of encephalomyelitis caused by Candida parapsilosis
Source: CNS Neurosci Ther. 2022 Dec 19;29(2):744–8. doi: 10.1111/cns.14062 (PMC9873509; doi:10.1111/cns.14062)

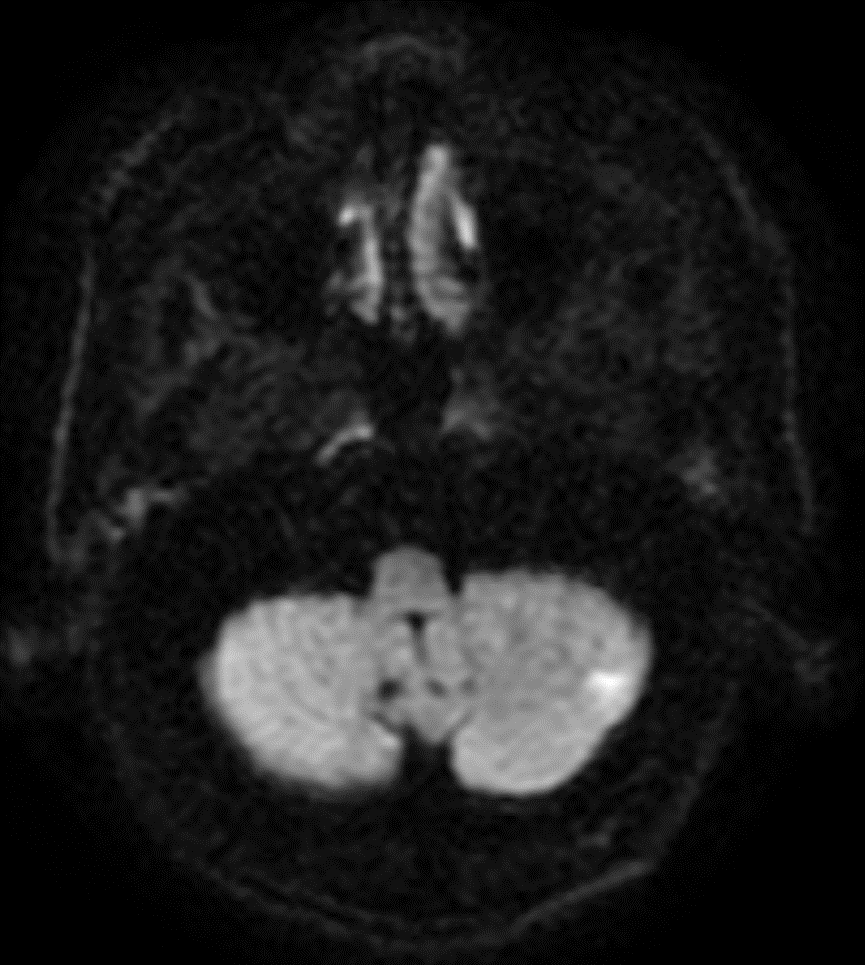


**Figure 1 A**


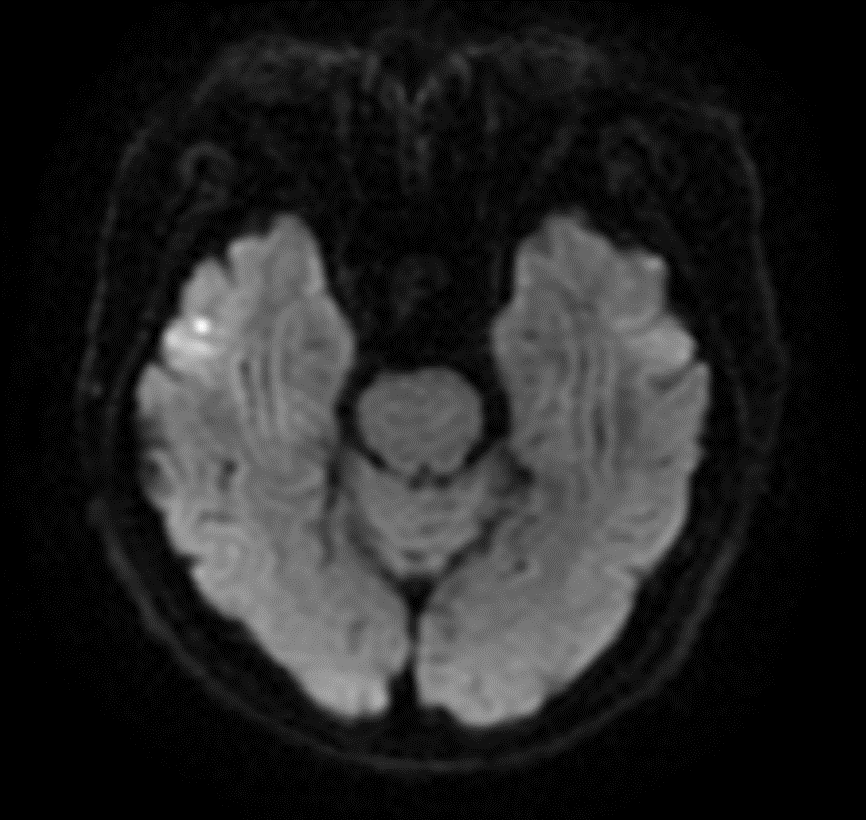


**Figure 1 B**


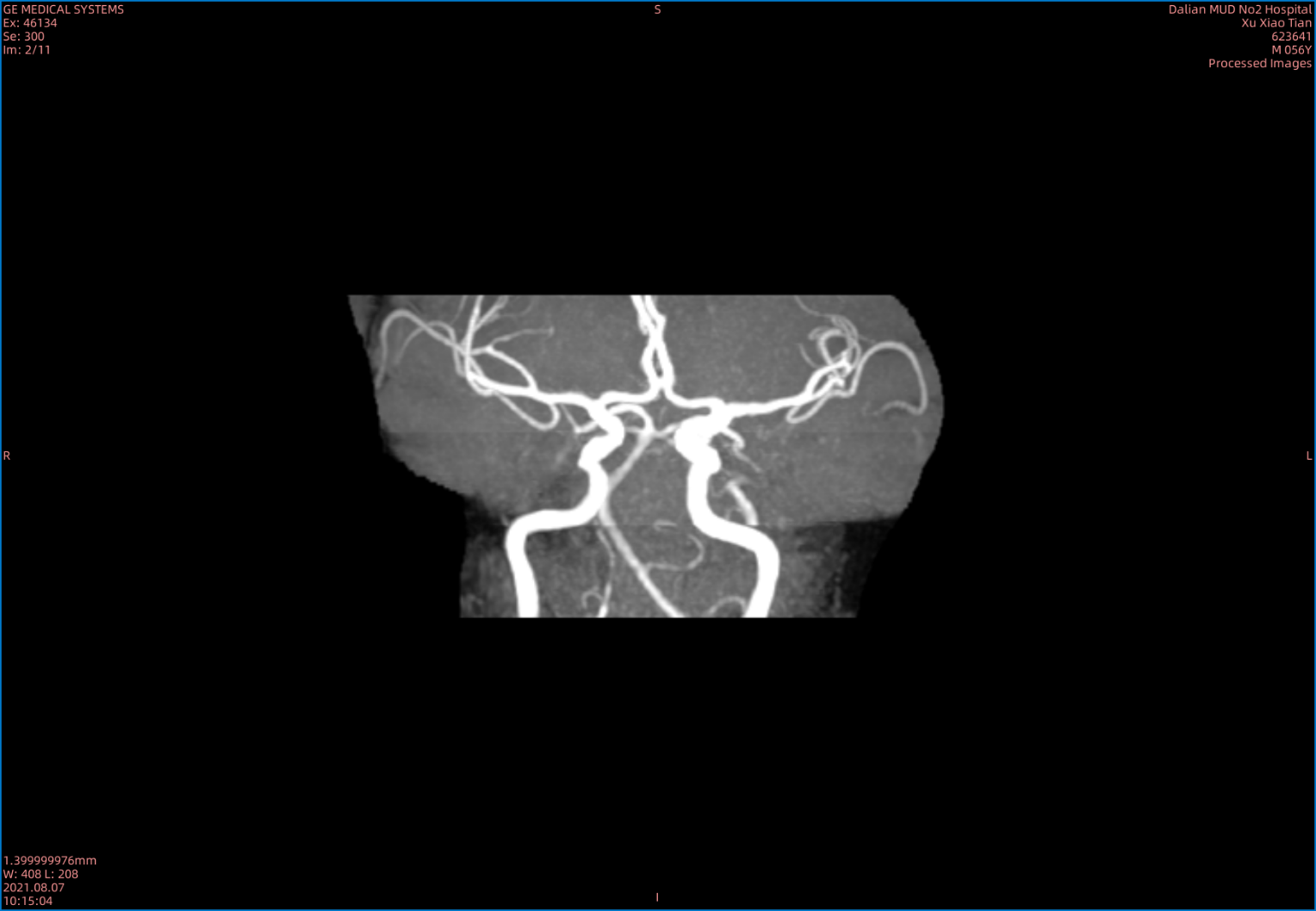


**Figure 1 C**


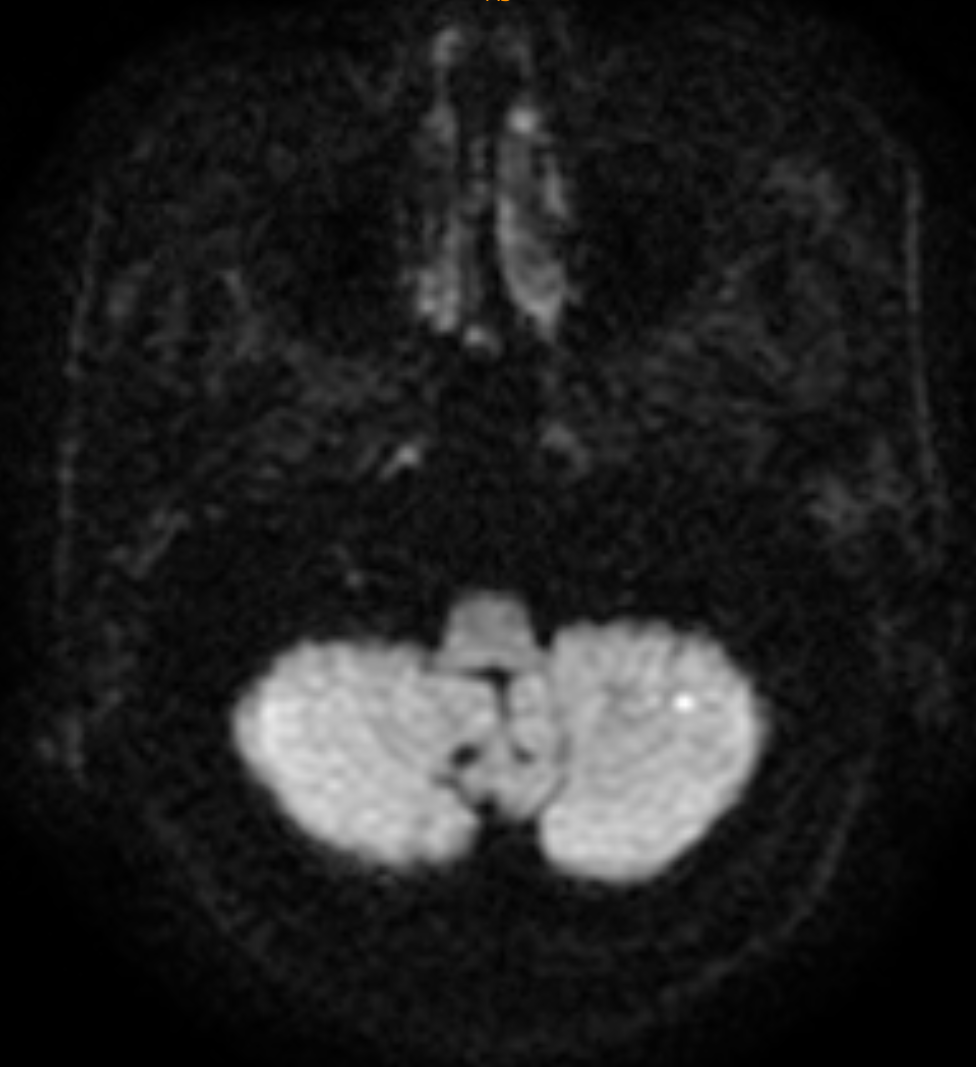


**Figure 1 D**


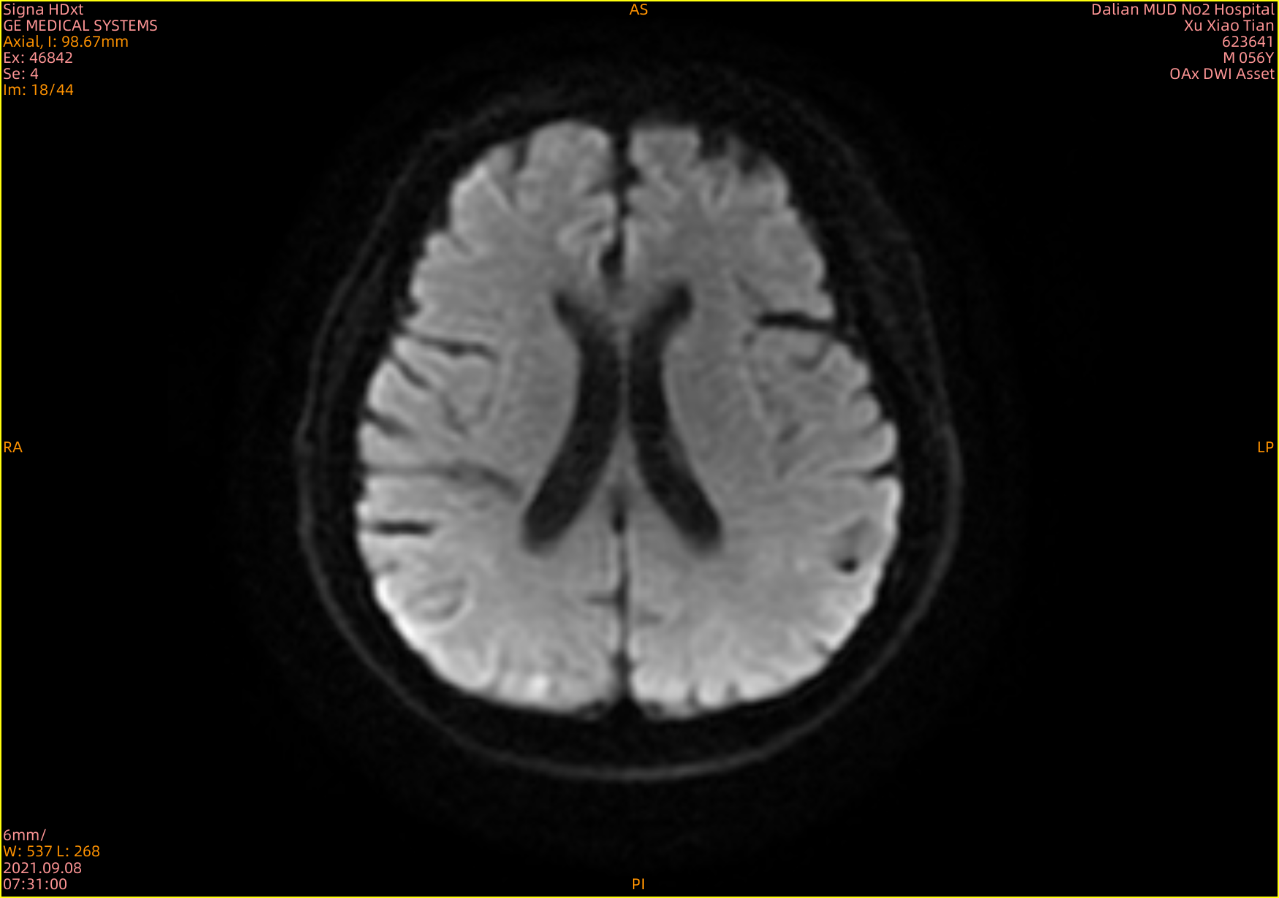


**Figure 1 E**


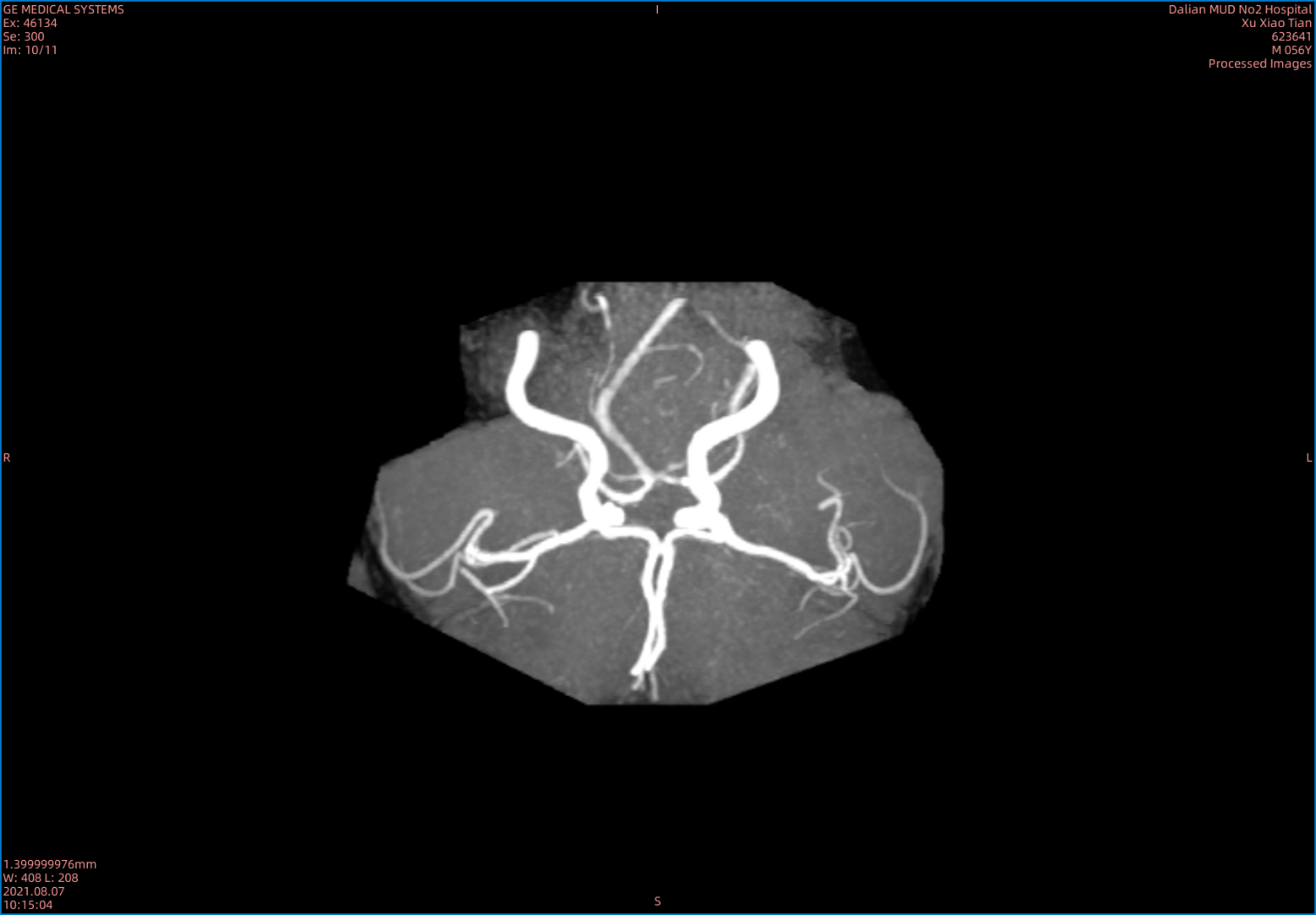


**Figure 1 F**


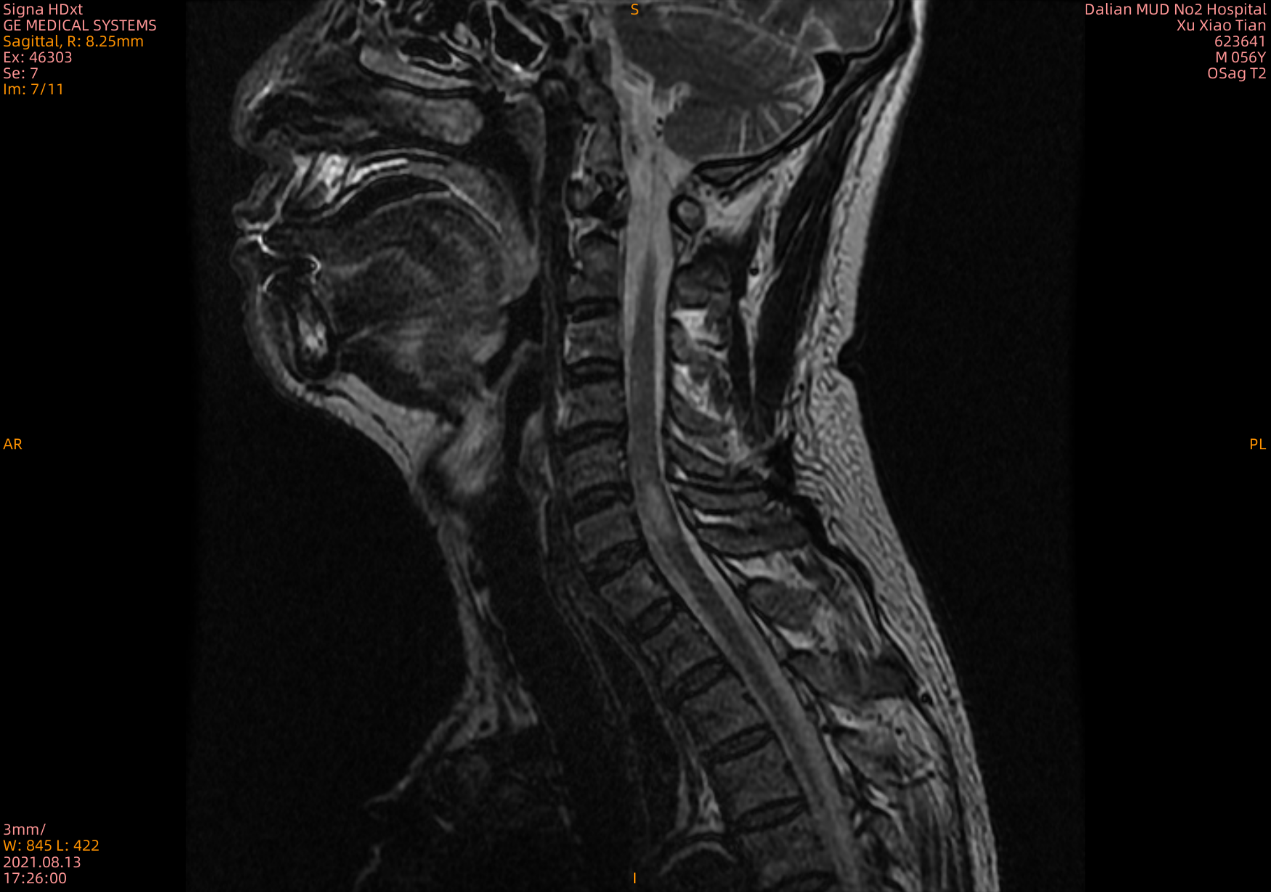


**Figure 2 A (1)**

**
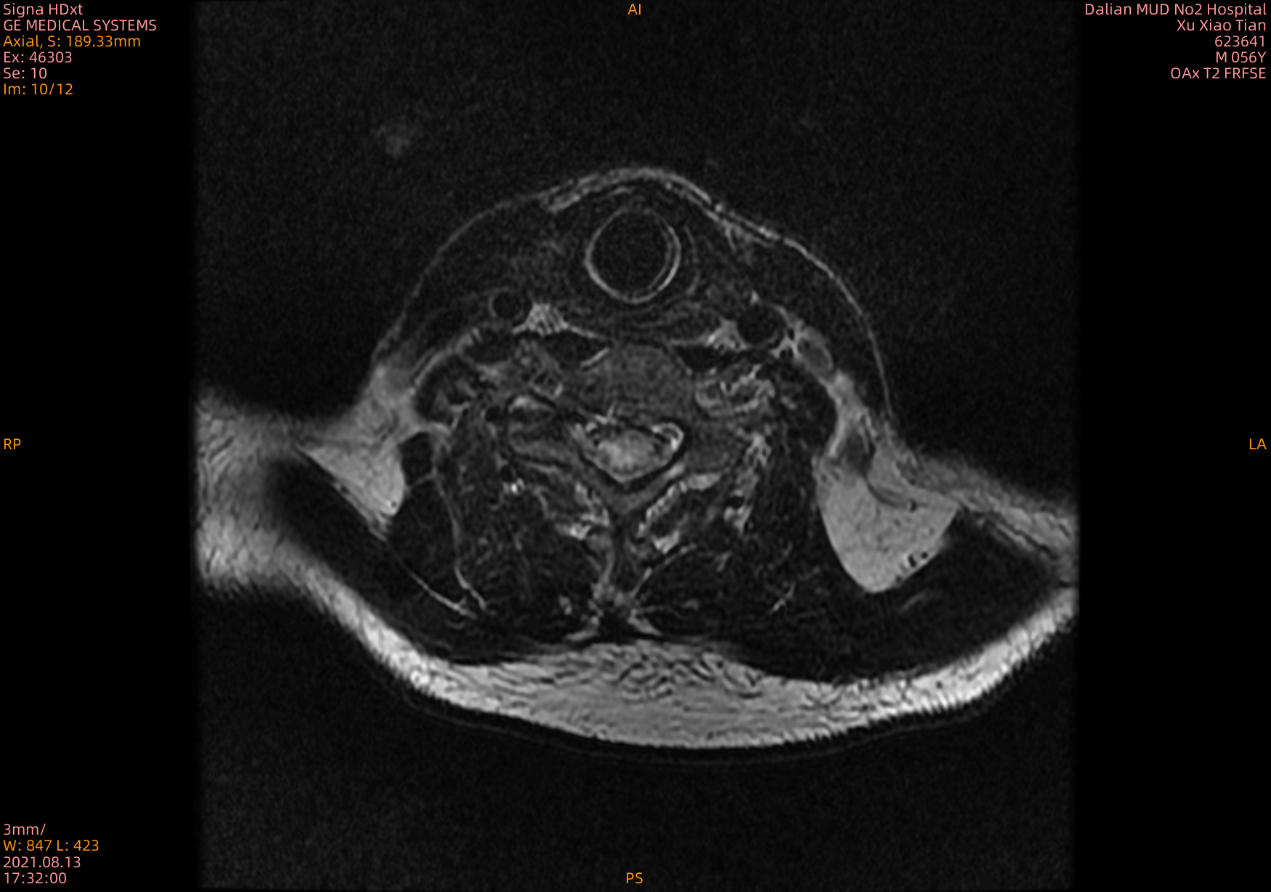
**

**Figure 2 A (2)**

**
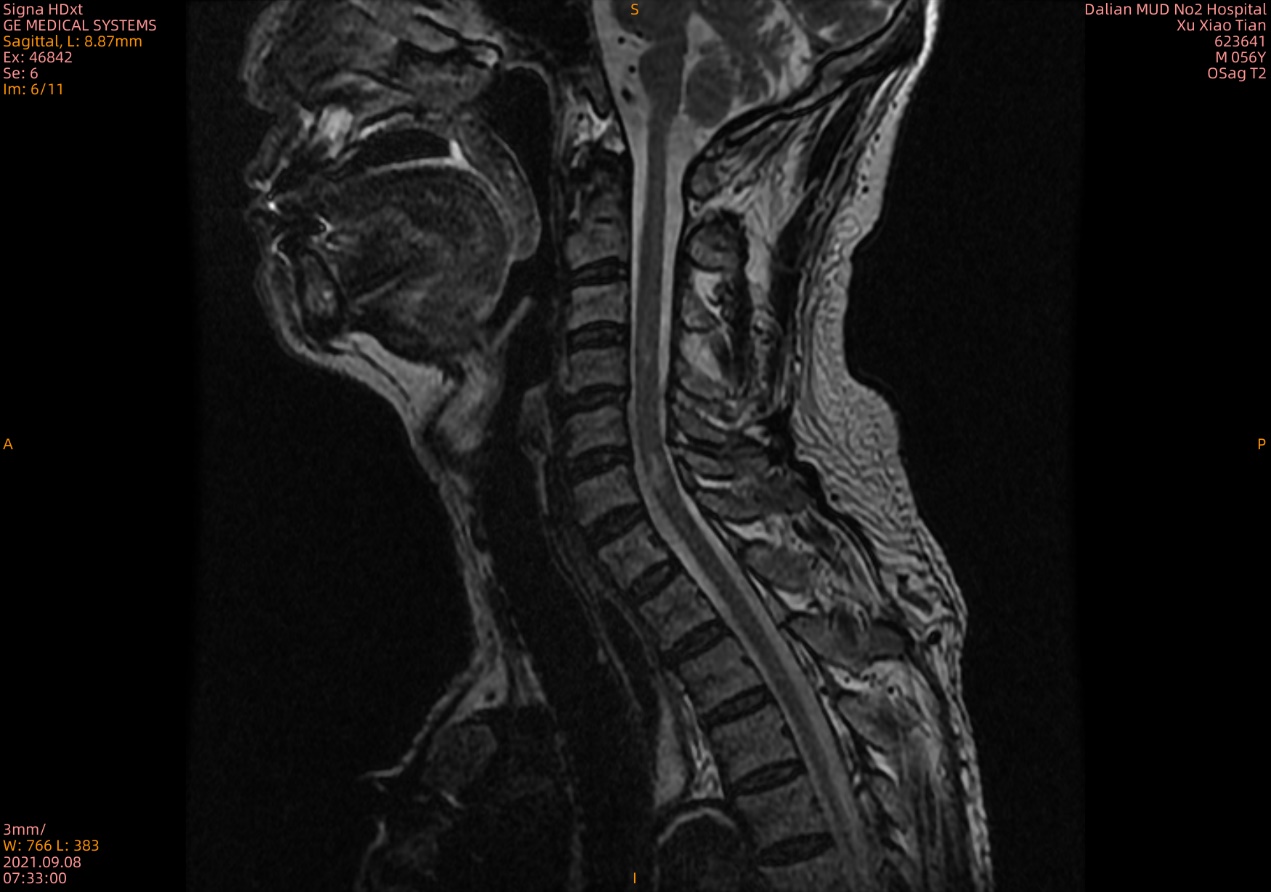
**

**Figure 2 B (1)**

**
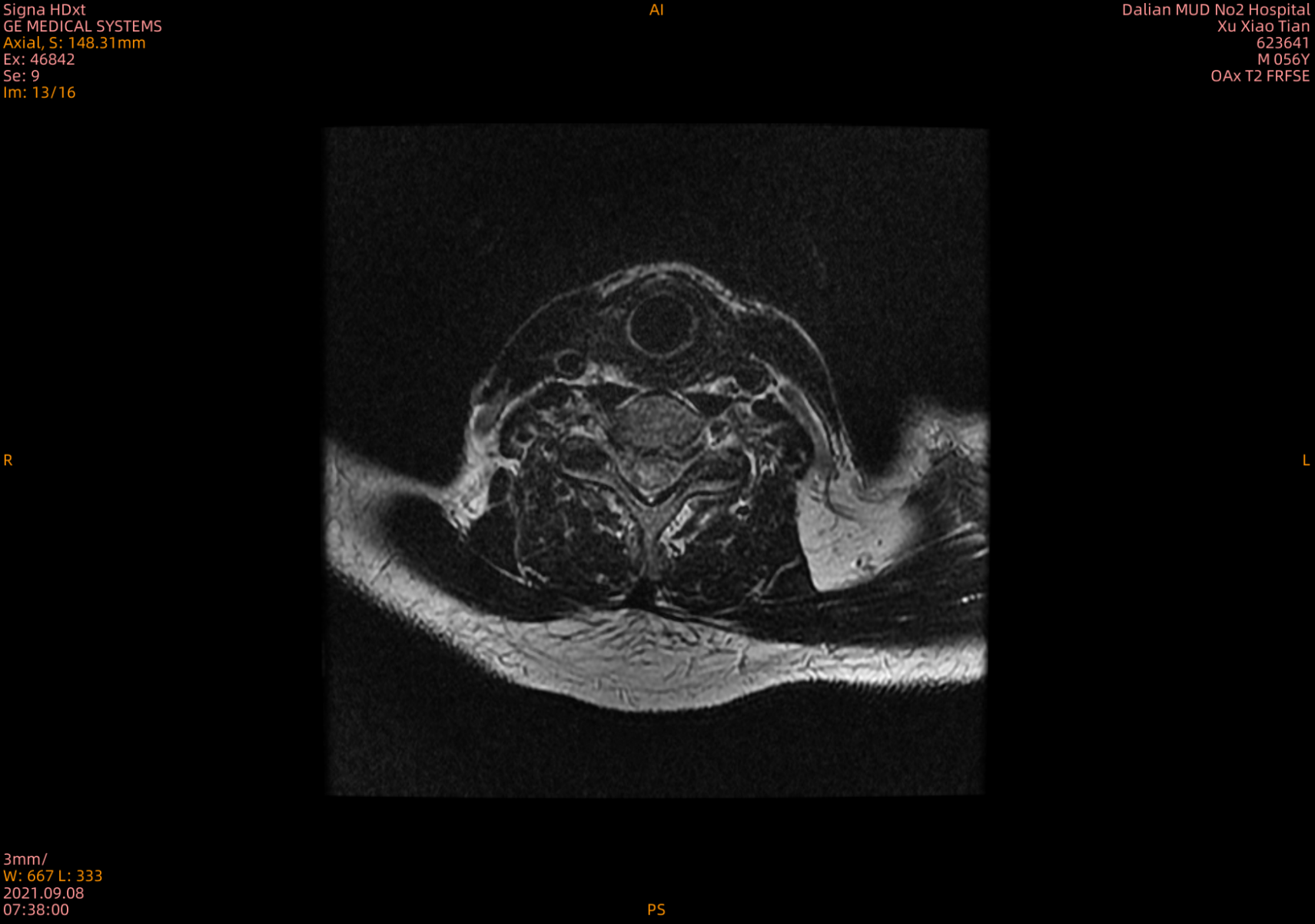
**

**Figure 2 B (2)**

Supplement: Supplementary file 1 — Appendix S1 [file CNS-29-744-s001.docx]
